# Picomolar Sensitivity Analysis of Multiple Bradykinin-Related Peptides in the Blood Plasma of Patients With Hereditary Angioedema in Remission: A Pilot Study

**DOI:** 10.3389/falgy.2022.837463

**Published:** 2022-02-11

**Authors:** François Marceau, Georges-Etienne Rivard, Jacques Hébert, Julie Gauthier, Hélène Bachelard, Tanja Gangnus, Bjoern B. Burckhardt

**Affiliations:** ^1^Axe Maladies Infectieuses et Immunitaires, Centre de Recherche du CHU de Québec-Université Laval, Québec, QC, Canada; ^2^Division of Hematology/Oncology, CHU Sainte-Justine, Université de Montréal, Montréal, QC, Canada; ^3^Service d'allergie, CHU de Québec-Université Laval, Québec, QC, Canada; ^4^Molecular Diagnostic Laboratory, Centre Hospitalier Universitaire Sainte-Justine, Université de Montréal, Montréal, QC, Canada; ^5^Department of Pediatrics, Centre Hospitalier Universitaire Sainte-Justine, Université de Montréal, Montréal, QC, Canada; ^6^Axe Endocrinologie et Néphrologie, Centre de Recherche du CHU de Québec-Université Laval, Québec, QC, Canada; ^7^Institute of Clinical Pharmacy and Pharmacotherapy, Heinrich Heine Universität Düsseldorf, Düsseldorf, Germany

**Keywords:** hereditary angioedema, bradykinin, *F12* variant, *SERPING1* variants, LC-MS/MS

## Abstract

**Background:**

Hereditary angioedema (HAE) is a rare autosomal dominant disease; the most well understood forms concern the haplodeficiency of C1 esterase inhibitor (C1INH) and a gain of function mutation of factor XII (FXII). The acute forms of these conditions are mediated by an excessive bradykinin (BK) formation by plasma kallikrein.

**Methods:**

A validated LC-MS/MS platform of picomolar sensitivity developed for the analysis of eleven bradykinin-related peptides was applied to the plasma of HAE-C1INH and HAE-FXII sampled during remission.

**Results:**

In HAE-C1INH plasma, the concentrations of the relatively stable BK_1−5_ fragment (mean ± S.E.M.: 12.0 ± 4.2 pmol/L), of BK_2−9_ (0.7 ± 0.2 pmol/L) and of the sums of BK and its tested fragments (18.0 ± 6.4 pmol/L) are significantly greater than those recorded in the plasma of healthy volunteers (1.9 ± 0.6, 0.03 ± 0.03 and 4.3 ± 0.8 pmol/L, respectively), consistent with the previous evidence of permanent plasma kallikrein activity in this disease. Kinin levels in the plasma of HAE-FXII patients did not differ from controls, suggesting that triggering factors for contact system activation are not active during remission.

**Conclusion:**

BK_1−5_, BK_2−9_ and the sum of BK and its fragments determined by the sensitive LC-MS/MS technique are proposed as potential biomarkers of HAE-C1INH in remission while this was not applicable to HAE-FXII patients.

## Introduction

Hereditary angioedema (HAE) is an autosomal dominant group of disorders that are determined by several gene variants proven or postulated to be permissive for bradykinin (BK) production, and possibly repressive for its degradation, with ensuing action on the endothelial BK B_2_ receptors and localized edema of subcutaneous and submucosal tissues (1, 2). Many variants of the *SERPING1* gene encoding C1 esterase inhibitor (C1INH) with impaired expression or function cause the most common form of HAE (types 1 and 2, respectively). A rarer form of HAE with normal C1-INH levels is caused by mutation of genes encoding coagulation factor XII (FXII, *F12* gene) (3); further, several other causal gene variants have been identified, some plausibly associated with the kallikrein-kinin system (plasminogen *PLG* and kininogen *KNG1*) (1). The most common variants of FXII causing HAE-FXII introduce new sites of cleavage by plasmin that accelerate cleavage by this protease, a basis for a gain of function in the contact system (4). However, for many patients, no mutation or abnormalities have been yet found and they may differ in several aspects including gender distribution, genetics, symptoms, and impact of estrogens.

The nonapeptide BK, formed by plasma kallikrein from high molecular kininogen (HK), is inherently unstable in blood plasma with a half-life in the order of 30 sec (5, 6). Several peptidases located in plasma or expressed at the surface of endothelial cells hydrolyze BK in live rats: in decreasing order of importance, angiotensin-I converting enzyme (ACE), aminopeptidase P, and neutral endopeptidase (7). Dipeptidyl peptidase IV and arginine carboxypeptidases have only marginal roles in the inactivation of BK *in vivo*. Single, repeated, or combined actions of these peptidases potentially generate many BK fragments, some of which were identified by high pressure liquid chromatography (HPLC) from concentrated synthetic BK incubated in plasma, serum or other peptidase sources (8, 9). Notably, the BK_1−5_ fragment, generated by 2 successive hydrolytic reactions catalyzed by ACE, is relatively stable and reportedly accumulates, notably after the infusion of synthetic BK in humans (10). Plasma BK_1−5_ also increases in acquired angioedema associated with therapeutic ACE inhibition (11), as BK breakdown inhibition may also result in angioedema.

Using a validated liquid chromatography coupled with tandem mass spectrometry (LC-MS/MS) platform of picomolar sensitivity developed for the analysis of eleven bradykinin-related peptides (12–16), we addressed the presence of BK-related peptides in the plasma of patients with HAE-C1INH and HAE-FXII during remission. We hypothesized that some of these peptides, including BK_1−5_, might be biomarkers of these forms of the disease, even if the buffering activity of kininases kept the patients symptom-free (kinin levels below a pharmacologic threshold for vascular effects).

## Materials and Methods

### Human Subjects

The local ethical review board (Comité d'éthique de la recherche, CHU de Québec-Université Laval) granted ethical approval to carry out the study involving blood donations from adult healthy volunteers and HAE patients 16 years old or older from the Province of Quebec, Canada (file no. 2022-6044). Locally recruited healthy volunteers also participated under the same ethical approval. All subjects gave written informed consent. Patient characteristics are listed in Tables 1, 2 and their blood was withdrawn when in remission. HAE patients received various prophylactic treatments that were not interrupted during the study (Table 2). Six female and one male patients heterozygotes for p.Thr328Lys in FXII (also known as T309K in reference to mature FXII sequence; 1032C>A in *F12*) were recruited. They were of Mediterranean descent. All HAE-FXII subjects, except the completely asymptomatic young male (subject F2), were included in a previous study (6); the molecular diagnostic procedures were described in that report. All HAE-FXII female patients, except one (subject F6), suffered from an essentially estrogen-dependent form of the disease, during pregnancies, oral contraception, or estrogen administration for menopausal symptoms. None of these conditions were present at the time of blood sampling. HAE related to *SERPING1* variants (haplodeficiency of C1INH) concerned well characterized patients diagnosed using low antigenic C4 levels, functional C1-INH measurements and familial history.

**Table 1:** Summary of the pilot study (all HAE patients seen in remission).

**Group**	**Age ±S.E.M**.	**Number of subjects**	**Number of female subjects**	Sum of BK and fragments ±S.E.M. (pmol/L) ^*^
Healthy volunteers	53.8 ± 4.7	9	7	4.3 ± 0.8
HAE-FXII	37.6 ± 4.7	7	6	7.4 ± 1.5
HAE-C1INH	50.1 ± 6.4	9	7	18.0 ± 6.3

**Figure 1:**
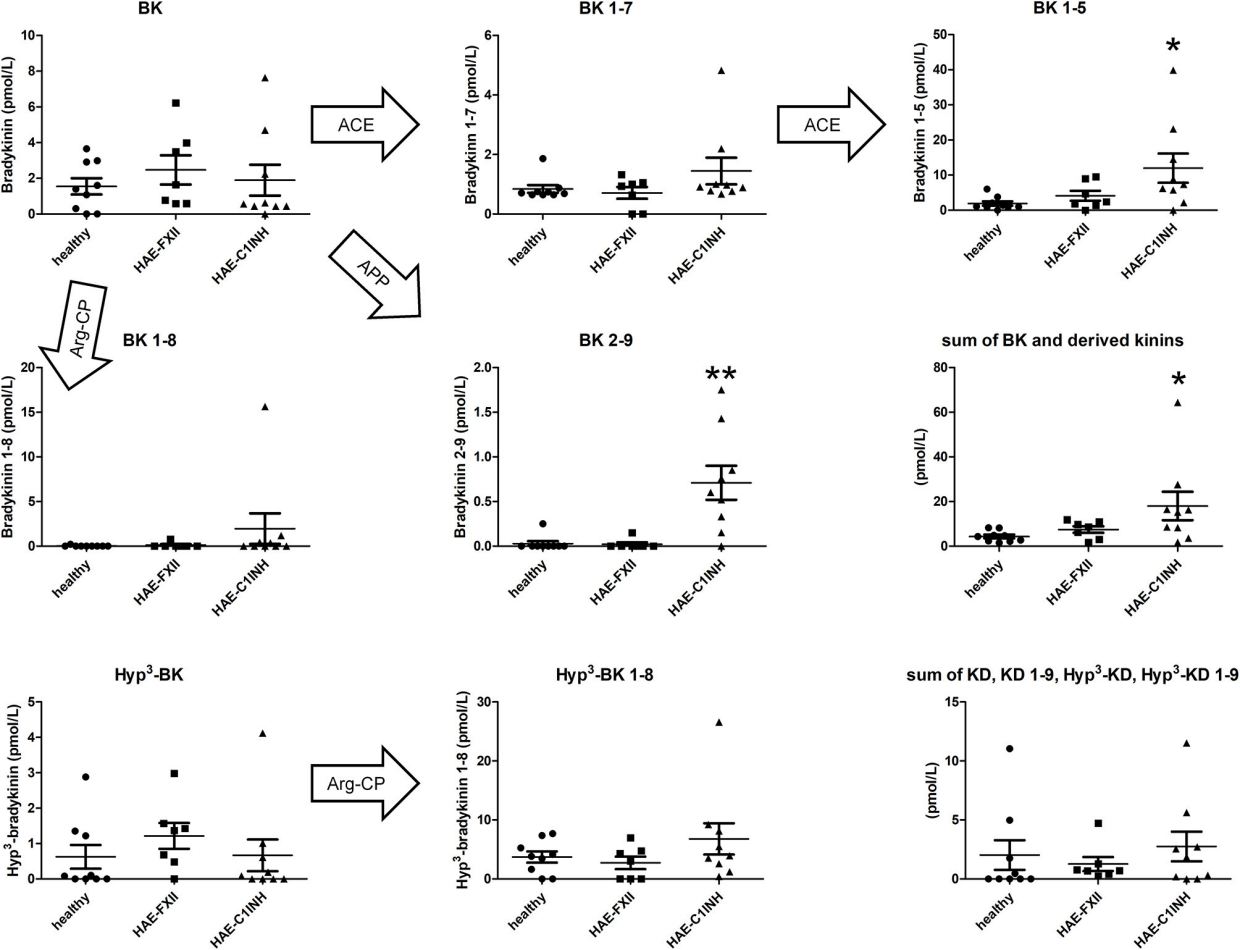
Top rows: concentration of various BK-related peptides determined in the plasma of venous blood from healthy volunteers or patients with HAE-FXII or HAE-C1INH in remission (demographic data in Table 1). An additional panel (second row, right) shows the sum of BK and its fragments for each individual, presumed to represent the minimal concentration of kinins derived from plasma kallikrein activity. Bottom row: concentration of additional kinins. Individual values are shown. Horizontal bars are the mean, and the intervals the S.E.M. The Kruskall-Wallis test was applied to compare the effect of diagnostic categories. When significant, Dunn's multiple comparison test was applied to compare the values from each type of HAE patients to those of the healthy controls. *P < 0.05; **P < 0.001. Arrows indicate metabolic derivation of peptides from BK or Hyp^3^-BK via the action of peptidases. ACE, angiotensin-I converting enzyme; APP, aminopeptidase P; Arg-CP, arginine carboxypeptidases; BK, bradykinin; Hyp, hydroxyproline; KD, kallidin (= Lys-BK); S.E.M., standard errors of the mean.

* *Statistics reported in Figure 1*.

**Table 2:** Characteristics of patients with HAE.

**No**.	**Sex, age range**	HAE type^*^	Prophylactic treatment^**^	**Last dose of prophylaxis before sampling**	**Sum of BK and fragments (pmol/L)**
H1	F, 61–65	1	Berinert	2 weeks	16.46
H2	F, 66–70	2	None		1.70
H3	F, 21–25	1	Haegarda	1 day	8.28
H4	M, 71–75	1	Berotralstat	14 h	64.39
H5	F, 26–30	1	Haergarda	1 day	3.54
H6	F, 51–55	1	Haegarda + Berinert	2 days	8.56
H7	M, 61–65	1	Berotralstat	14 h	27.56
H8	F, 41–45	1	None		16.20
H9	F, 31–35	1	Berinert	2 days	15.17
F1	F, 46–50	*F12* T328K	Tranexamic acid	2 h	11.82
F2^***^	M, 16–20	*F12* T328K	None		1.60
F3	F, 41–45	*F12* T328K	None		10.88
F4	F, 26–30	*F12* T328K	None		9.72
F5	F, 36–40	*F12* T328K	None		8.62
F6^****^	F, 51–55	*F12* T328K	None		6.23
F7	F, 36–40	*F12* T328K	None		3.05

* *Types 1 and 2 are variants of C1INH haplodeficiencies*.

** *Berinert and Haegarda are C1INH concentrates*.

*** *Son of F1*.

**** *mother of F4, F5, F7*.

### Blood Sampling for Kinin Analysis

Blood of adult healthy volunteers and HAE patients was sampled in protease-inhibitor prespiked EDTA S-Monovettes® (Sarstedt, Nümbrecht, Germany). The applied seven-component protease inhibitor was previously shown to efficiently stabilize kinin levels *ex vivo* (14). In addition, a standardized procedure was applied for blood sampling to control impacts of blood sampling on generation of BK levels (16). In short, blood was sampled via butterfly needles with the aspiration technique. After blood sampling, kinins were centrifuged within 30 min and plasma was frozen and stored at −80 °C until analysis.

### LC-MS/MS Determination of Kinins

Kinins were measured based on a validated LC-MS/MS platform (15) being extended specifically for the present study. In comparison, an enhanced lower limit of quantification was achieved and the extend of measured kinins was increased to eleven (BK, BK_1−8_, BK_2−9_, BK_1−7_, BK_1−5_, Hyp^3^-BK, Hyp^3^-BK_1−8_, KD, KD_1−9_, Hyp^4^-KD, Hyp^4^-KD_1−9_). To prove the validity and collection of high-quality data, all measurements were handled within a quality control system. The determination was conducted according to Good Clinical Laboratory Practice. Concentrations of unknown samples were reported up to the detection limit; values below this threshold were set to zero.

### Data Analysis

Numerical values are reported as means ± standard errors of the mean (S.E.M.), a distribution-free assessment of mean value uncertainty. Sets of values were compared with Kruskall-Wallis test (non-parametric ANOVA) followed by Dunn's multiple comparison test to compare selected pairs of values (Prism 5.0, GraphPad Software Inc., San Diego, CA).

## Results

Three groups of subjects were included in this pilot study: healthy volunteers and patients with HAE-FXII or HAE-C1INH (demographic data reported in Table 1). The concentrations of kinin peptides measured in the plasma of venous blood samples are illustrated in Figure 1 for the 3 groups of subjects. The physiopathology of both forms of HAE involves plasma kallikrein that directly releases BK from HK (2). BK concentrations in all three groups are extremely low in the samples (<10 pmol/L, Figure 1), consistent with its short half life and catabolism by multiple peptidases. This finding is in line with measured values in a larger cohort of 24 healthy adults using the same LC-MS/MS method (data not yet published). Low levels were further observed for the primary BK metabolite generated by ACE, BK_1−7_. However, BK_1−5_, produced by a further reaction of BK_1−7_ with ACE, has a better stability (8–10) and is a potential biomarker of HAE-C1INH, as its concentration is significantly higher in this group than in controls (Figure 1). On the other hand, BK_1−5_ concentrations in HAE-FXII plasma did not differ significantly from those of healthy volunteers.

The metabolites of BK generated by alternate peptidases were examined. Arginine carboxypeptidases (carboxypeptidases N, M) produce BK_1−8_; this peptide was not informative in the present study (Figure 1). Aminopeptidase P is the second-most important BK clearing peptidase *in vivo*, after ACE (7). The concentration of the resulting BK_2−9_ fragment, itself biologically inactive (17), is nevertheless the most discriminative biomarker of HAE-C1INH in the present study despite its very low concentration (Figure 1). BK_2−9_ remains mostly undetectable in the plasma of HAE-FXII patients. A further analysis was attempted by adding up the non-overlapping concentrations of BK, BK_1−7_, BK_1−5_, BK_1−8_ and BK_2−9_, based on the assumption that their sum may represent the minimal kinin concentration generated by plasma kallikrein, despite their different half-life and the limited selection of peptides. The sums are significantly higher in the HAE-C1INH patients than in healthy volunteers, the values from HAE-FXII patients remaining similar to those of controls (Figure 1, Table 1).

A fair proportion of kininogen molecules in the circulation are affected by a post-translational hydroxylation of the proline residue that coincide with Pro^3^ in the BK sequence (Pro^4^ in kallidin = Lys-BK) (18, 19). Whether the hydroxyproline (Hyp)-containing kinin are preferentially released from either form of kininogens by either form of kallikreins is not clear at present, but the pharmacology of Hyp^3^-BK does not quantitatively differ from that of BK for interaction with the human B_2_ receptor (20, 21). However, the applied LC-MS/MS technique readily differentiate kinins possessing the Hyp residue owing to a different molecular weight. Validated tests for Hyp^3^-BK and Hyp^3^-BK_1−8_ showed no differences between the 3 groups of subjects (Figure 1). Measurements of Hyp^3^-BK_1−5_ or Hyp^3^-BK_2−9_, possibly more discriminative, were not available.

Kallidin (KD = Lys-BK) is formed by the action of tissue kallikrein, mostly on low molecular weight kininogen (22). It is not believed that HAE-FXII or HAE-C1INH involve tissue kallikrein; rather, plasma kallikrein is a validated target for the development of prophylactic drugs such as lanadelumab, berotralstat and ecallantide for the prophylaxis of classical HAE attacks (23, 24). KD, Hyp^4^-KD and their 1-9 fragments (with Arg^10^ removed) were measured in the plasma of the 3 groups of subjects. Concentrations generally remained very low and independent of the diagnostic category (the sums of the 4 peptides are illustrated in Figure 1).

Examination of sex or prophylactic treatments did not provide obvious explanations for extreme values of kinin concentrations in the patients with HAE group (Table 2 reports individual values of the sum of BK and fragments).

## Discussion

Technological developments based on LC-MS/MS now supports the detection of several kinins with picomolar sensitivity and high reproducibility in biological fluids (12–16). The inclusion of biologically inactive fragments allows addressing the relative effects of various peptidases known to hydrolyze BK-related peptides. The presence of cleaved HK in the blood of HAE-C1INH is well established during remission (25, 26) as well as a spontaneous plasma kallikrein activity (measured as the hydrolysis of synthetic HD-Pro-Phe-Arg-*p*NA) (27, 28). These results suggest a continuous formation of BK during remission in HAE-C1INH. While kinins are short-lived mediators *in vivo*, we hypothesized that the exquisitely sensitive LC-MS/MS detection of kinins might pick up bioactive kinins and their metabolites during remission of HAE-C1INH in a small sample of patients mostly under pharmacologic attack prophylaxis. It is a tribute to the detoxifying of multiple catabolic pathways that kinin concentrations in the venous blood were exceedingly small in all groups; even in the HAE-C1INH patients, the sum of BK and its fragments remained below a threshold for a biological activity mediated by the BK B_2_ receptors. The concentration of the relatively stable metabolite BK_1−5_ generated from BK by ACE was significantly higher in these patients than that recorded in healthy volunteers, albeit with some overlap. The summed concentration of BK and its fragments was also significantly higher (Figure 1). An unexpected result was that the BK_2−9_ fragment, generated from BK by aminopeptidase P in a single step, behaved as a potential discriminative biomarker of HAE-C1INH, although the concentrations of BK_2−9_ were low in absolute values.

A relatively common form of HAE with normal C1INH involves a mutated FXII; the T328K or T329R substitutions introduce a new cleavage site for plasmin (4) and perhaps thrombin (29). Uncontrolled activation of plasma kallikrein is the consequence of this unconventional FXII cleavage. Although spontaneous cleavage of HK was anecdotally reported in HAE-FXII during remission (30), the plasma kallikrein enzymatic activity generally remained low; however, this activity definitely increased during attacks of HAE-FXII (27). We did not evidence increased concentration of kinin peptides in HAE-FXII patients in remission (Figure 1) and this must reflect the absence of triggering factors for the activation of the kallikrein-kinin system. The activation of the T328K mutant of FXII is postulated to occur during coagulation and fibrinolysis (4, 29), irrelevant processes in our patient sample, as well as the influence of estrogens. Indeed, attacks of HAE-FXII are particularly determined by the hormonal status, very rare in males and associated with estrogens as in pregnancies, oral contraception, and hormonal supplement for menopausal symptoms (31). On note, one of our female patients (subject F6) was never affected by attacks, although her 3 daughters were symptomatic in an estrogen-dependent manner in the past. These patients were included in a previous study where the patients' citrated plasma was incubated *ex vivo* and stimulated with recombinant tissue plasminogen activator to activate plasmin. Relative to control plasma, an explosive and rapid production of BK was observed in plasmas from all HAE-FXII patients including F6 (enzyme immunoassay of BK corroborated with signaling measurements in cells that expressed the human B_2_ receptors) (6). These sharp differences in laboratory findings suggest that assessing *ex vivo* kinin formation is a promising complementary approach to investigate HAE with normal level of C1INH, especially if the very nuanced LC-MS/MS technique is exploited to quantify multiple kinin peptides.

KD is generated by tissue kallikrein (22); this form of secreted kallikrein is not relevant to the contact system and, therefore, to the physiopathology of the examined types of HAE. Of interest, the metabolite of KD generated by arginine carboxypeptidases, KD_1−9_ (or Lys-des-Arg^9^-BK) is the optimal agonist of the human kinin B_1_ receptor (32). The sum of KD and its fragments remained low and uninfluenced by HAE (Figure 1); such peptides may derive from alternate physiological or pathological processes. KD and KD_1−9_ were identified in nasal lavage fluid collected in healthy volunteers (13). However, the removal of the N-terminal Lys residue from these peptides by aminopeptidase N (CD13) (33) could potentially “contaminate” the concentration values of BK and its fragments to a small extent.

The limitations of the present pilot study are that the number of subjects is small, that the HAE patients were not seen during attacks and that many were under prophylactic treatments. Further, the detection of the most relevant fragments of Hyp^3^-BK were not presently clinically validated.

In conclusion, the concentrations of BK_1−5_, BK_2−9_ and the sum of BK and its fragments determined by a sensitive LC-MS/MS technique are proposed as potential biomarkers of HAE-C1INH in remission. This was not applicable to HAE-FXII, although excessive stimulated *ex vivo* BK generation was previously demonstrated. Future work based on the LC-MS/MS technique applied to *ex vivo* generation of multiple kinin peptides by standardized stimuli (6, 34, 35) is warranted to address the physiopathology of other HAE forms with normal C1INH level, especially because the spontaneous activity of tissue kallikrein is often low in these patients (27). The detection of multiple kinin metabolites is also ideal to address the effect of genetically determined deficiencies in kininase expression, postulated to interact with other causal genes for the severity of HAE states (1, 36).

## Data Availability Statement

The original contributions presented in the study are included in the article/supplementary material, further inquiries can be directed to the corresponding author/s.

## Ethics Statement

This study received approval by the local Ethical Review Board (Comité d'éthique de la recherche, CHU de Québec-Université Laval, file no. 2022-6044). Written informed consent to participate in this study was provided by the participants or their legal guardian/next of kin.

## Author Contributions

G-ER, JH, and FM identified and interacted with patients and organized sampling. TG, BB, FM, G-ER, JG, and HB participated to the experimental work. FM analyzed results and wrote the manuscript draft. All authors read and approved the final manuscript.

## Funding

Supported by the patient association Angio-Œdème Héréditaire du Québec.

## Conflict of Interest

FM served as a consultant for Pharvaris B.V. and received research funds from Shire/Takeda and Pharvaris B.V., outside the submitted work. G-ER has been a member of advisory boards (Baxalta, Bayer, Biogen Idec, CSL Berhing, Novo Nordisk, Octapharma, Pfizer) and received funding from Bayer, CSL Behring and Pfizer (unrelated to the submitted work). JH has been a speaker/teacher for CLS Behring, Novartis, and Shire; he has been a member of advisory committees (CLS Behring, Shire, and Novartis) and a clinical investigator for Merck (ALK), Stallergene, Boehringer-Ingelheim, GlaxoSmithKline (GSK), Novartis, Sanofi, AstraZeneca, CLS Behring, Shire, Roche, and Griffols (unrelated to the submitted work). The remaining authors declare that the research was conducted in the absence of any commercial or financial relationships that could be construed as a potential conflict of interest.

## Publisher's Note

All claims expressed in this article are solely those of the authors and do not necessarily represent those of their affiliated organizations, or those of the publisher, the editors and the reviewers. Any product that may be evaluated in this article, or claim that may be made by its manufacturer, is not guaranteed or endorsed by the publisher.
